# Counteractive and cooperative actions of muscle β-catenin and Ca_V_1.1 during early neuromuscular synapse formation

**DOI:** 10.1016/j.isci.2022.104025

**Published:** 2022-03-04

**Authors:** Mehmet Mahsum Kaplan, Bernhard E. Flucher

**Affiliations:** 1 Department of Physiology and Medical Physics, Medical University Innsbruck, 6020 Innsbruck, Austria

**Keywords:** Biological sciences, Developmental neuroscience, Cell biology, Developmental biology

## Abstract

Activity-dependent calcium signals in developing muscle play a crucial role in neuromuscular junction (NMJ) formation. However, its downstream effectors and interactions with other regulators of pre- and postsynaptic differentiation are poorly understood. Here, we demonstrate that the skeletal muscle calcium channel Ca_V_1.1 and β-catenin interact in various ways to control NMJ development. They differentially regulate nerve branching and presynaptic innervation patterns during the initial phase of NMJ formation. Conversely, they cooperate in regulating postsynaptic AChR clustering, synapse formation, and the proper organization of muscle fibers in mouse diaphragm. Ca_V_1.1 does not directly regulate β-catenin expression but differentially controls the activity of its transcriptional co-regulators TCF/Lef and YAP. These findings suggest a crosstalk between Ca_V_1.1 and β-catenin in the activity-dependent transcriptional regulation of genes involved in specific pre- and postsynaptic aspects of NMJ formation.

## Highlights

• Neuromuscular junction formation requires either muscle calcium or β-catenin signaling
• Complementary actions of Ca_V_1.1 and β-catenin control presynaptic innervation patterns
• Parallel actions of Ca_V_1.1 and β-catenin are crucial for postsynaptic AChR clustering
• Loss of Ca_V_1.1 differentially regulates activity of β-catenin targets TCF/Lef and YAP

## Graphical abstract

**Figure:**
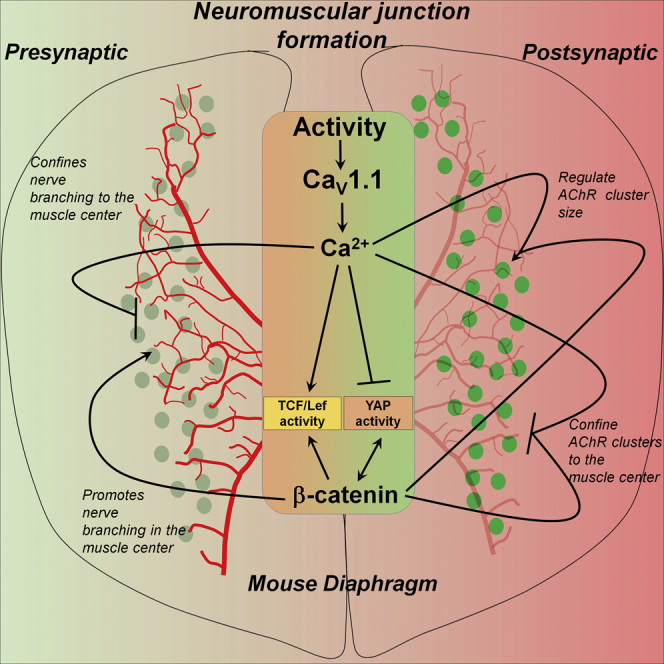

## Introduction

Regulation of neuromuscular synaptogenesis is a complex process in which multiple signaling pathways cooperate in the precise formation of these nerve-muscle connections (Sanes and Lichtman, 2001; Burden et al., 2018; Li et al., 2018). Although presynaptic nerve-derived factors, such as agrin and ACh, regulate clustering or dispersal of AChRs, and ultimately the patterning and stabilization of postsynaptic AChR clusters (Reist et al., 1992; Gautam et al., 1996; Glass et al., 1996; Misgeld et al., 2002, 2005), muscle-intrinsic mechanisms are now well appreciated to initiate not only postsynaptic differentiation (Lin et al., 2001; Yang et al., 2001) but also multiple aspects of presynaptic differentiation (Rafuse et al., 2000; Schwander et al., 2004; Fox et al., 2007; Li et al., 2008; Wu et al., 2012a, 2012b; Liu et al., 2012; Yumoto et al., 2012). During NMJ formation, AChRs are expressed and clustered in the central muscle domain independently of the motor nerves, thus defining the prospective innervation territory. Motor nerves follow a stereotypical branching pattern, as they approach this innervation territory, recognize their targets, and establish contacts with pre-formed AChR clusters. Importantly, muscle-derived signals induce the proper differentiation of the nerve terminals (Li et al., 2018). Both, the skeletal muscle calcium channel Ca_V_1.1 (Powell et al., 1984; Chen et al., 2011; Kaplan et al., 2018; Kaplan and Flucher, 2019) and the cytoplasmic signaling protein β-catenin (Li et al., 2008) play pivotal roles in these early steps of NMJ formation, representing two key controllers of pre- and postsynaptic development at the NMJ. However, a communication between Ca_V_1.1- and β-catenin-driven mechanisms hitherto has not been identified.

Ca_V_1.1, also known as dihydropyridine receptor (DHPR), is a member of the L-type calcium channel family, whose expression is specific to skeletal muscle (Bannister and Beam, 2013). Its primary differentiated function is that of the voltage sensor for skeletal muscle excitation-contraction coupling (Tanabe et al., 1988). In addition, recent evidence identified Ca_V_1.1 as a crucial regulator of NMJ formation downstream of synaptic and electrical activity (Chen et al., 2011; Kaplan et al., 2018; Kaplan and Flucher, 2019). In mice deficient of Ca_V_1.1, NMJ development is severely perturbed in that AChR clusters fail to form a narrow central endplate band (Powell et al., 1984; Chen et al., 2011; Kaplan et al., 2018), nerve trunks are miss-localized and defasciculated, and nerve terminals fail to correctly differentiate (Kaplan and Flucher, 2019). However, how Ca_V_1.1 translates muscle electrical activity into intracellular mechanisms controlling pre- and postsynaptic differentiation *in vivo* remained elusive.

An elegant study by Li et al. (2008) identified a critical role of muscle β-catenin in the retrograde regulation of motor nerve differentiation (Li et al., 2008). In diaphragm muscles lacking β-catenin, both postsynaptic and presynaptic differentiation are distorted. AChR clusters become distributed over a broad region, nerve trunks are miss-localized and show fewer secondary branches, and few synaptic vesicles accumulate in the nerve terminals, altogether resulting in functional defects. On the other hand, β-catenin gain-of-function mutations lead to increased motor nerve branching (Wu et al., 2012b; Liu et al., 2012). The role of β-catenin in NMJ formation was shown to be dependent on its transcriptional rather than cell adhesion activity (Wu et al., 2015). Expression of Slit2 in muscle is regulated by β-catenin, and Slit-Robo signaling was identified as one of the retrograde pathways implicated in these aspects of NMJ formation (Wu et al., 2015). In fact, mice lacking Slit2 show striking defects of motor nerve fasciculation and branching patterns (Jaworski and Tessier-Lavigne, 2012), similar to muscle-specific β-catenin knockout mice. Although, overexpression of Slit2 in muscle-specific β-catenin knockout mice reversed only nerve terminal defects, but not fasciculation and branching defects, indicating the necessity of additional retrograde mechanisms (Wu et al., 2015).

The striking parallels in the presynaptic phenotypes in Ca_V_1.1^−/−^ mice and on muscle-specific knockout of β-catenin, specifically the unrestricted nerve defasciculation and the presence of an ectopic nerve trunk, suggested a crosstalk between β-catenin- and Ca_V_1.1-regulated retrograde mechanisms (Kaplan and Flucher, 2019; Liu et al., 2019). Here, we show that knockout of β-catenin partially rescues the aberrant motor nerve branching in HSA-β-cat^−/−^; Ca_V_1.1^−/−^ double-mutant mice. The innervation patterns in single and double knockouts indicate a complementary activity of muscle β-catenin and Ca_V_1.1 in nerve branching. In contrast, AChR clustering was more severely compromised in double knockouts compared to each of the individual knockouts, indicative of a synergistic role in postsynaptic differentiation. Our results further indicate that Ca_V_1.1 does not directly regulate β-catenin expression, but acts through its transcriptional co-activators TCF/Lef and YAP. Together, the data indicate that muscle β-catenin and Ca_V_1.1 work together by (a) antagonistically controlling presynaptic motor nerve branching and (b) cooperatively controlling postsynaptic AChR clustering and (c) that parallel acting Ca_V_1.1 and β-catenin signaling pathways converge at the level of transcriptional regulators.

## Results

### Motor nerve innervation pattern is determined by opposite but complementary functions of Ca_V_1.1 and β-catenin

**Figure 1:**
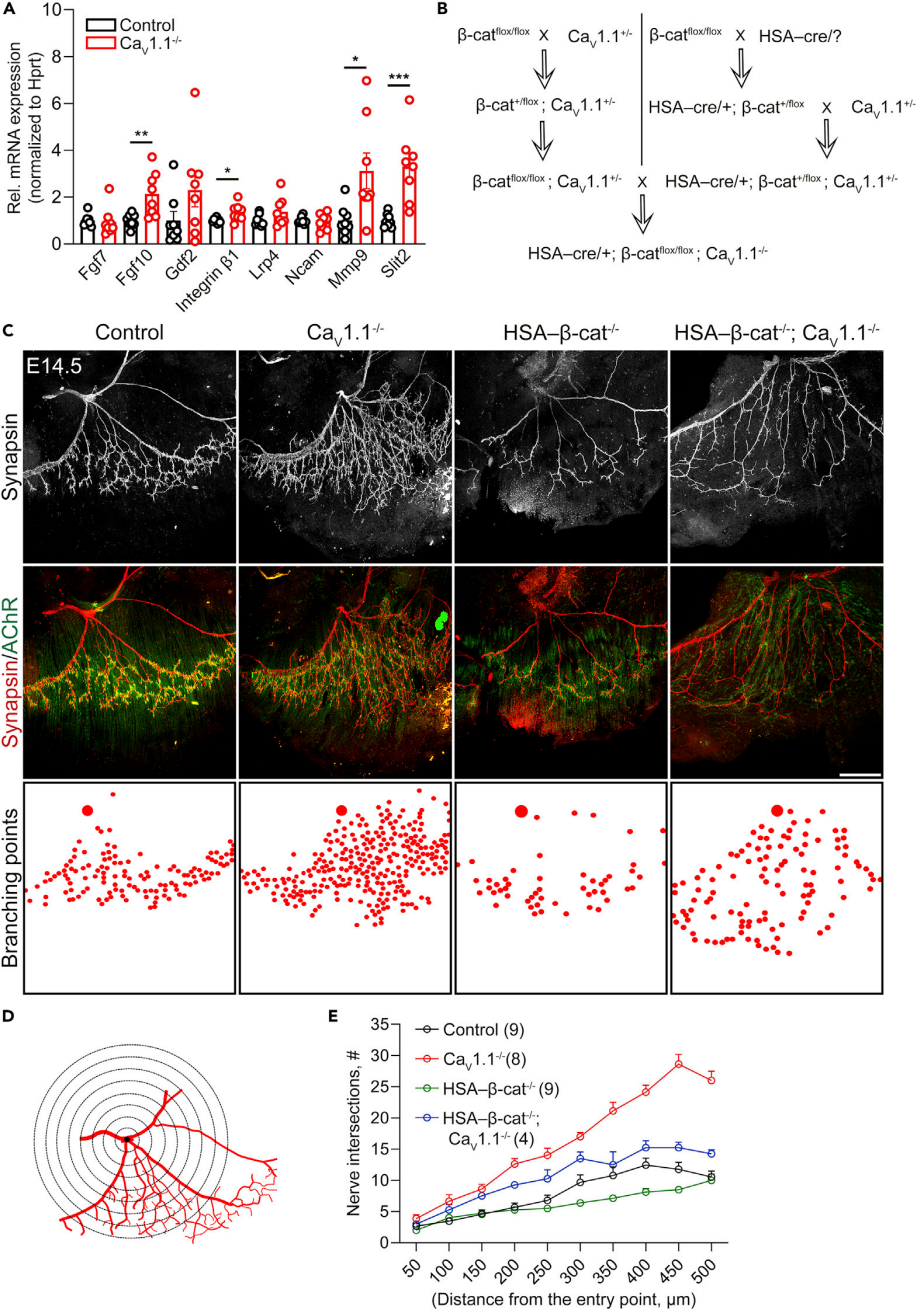
Complementary roles of Ca_V_1.1 and β-catenin in shaping the innervation pattern in E14.5 diaphragms (A) qRT-PCR analysis of mRNAs of putative retrograde signals from diaphragm lysates of E18.5 Ca_V_1.1^−/−^ mice and their control littermates shows increased expression levels of Fgf10, Integrin β1, Mmp9, and Slit2 in diaphragms lacking Ca_V_1.1. N = 3 litters, 8 mice. mean ± SEM; t-test, ∗p = 0.0185, ∗∗p = 0.0059, ∗∗∗p = 0.0007. (B) Breeding scheme used to generate muscle-specific β-catenin knockout mice in the Ca_V_1.1^−/−^ background. (C) Whole-mount right diaphragm preparations stained with anti-synapsin (red) and α-BTX (green) at E14.5 to label motor nerve branches and AChR clusters, respectively, in control, Ca_V_1.1^−/−^, HSA-β-cat^−/−^, and HSA-β-cat^−/−^; Ca_V_1.1^−/−^ mice. In Ca_V_1.1^−/−^ mice, motor nerve branching occurs throughout the diaphragm. In HSA-β-cat^−/−^ mice, branching is reduced compared to controls and confined to the central muscle domain. HSA-β-cat^−/−^; Ca_V_1.1^−/−^ double-knockout mice display motor nerve branching throughout the diaphragm, however, at a substantially decreased extent compared to Ca_V_1.1^−/−^ mice. Branching points are indicated in boxes below with red dots. Scale bar, 300 μm. (D) Schematic demonstration of Sholl analysis applied to quantify motor nerve branching using the entry point of the nerve into the diaphragm as origin. (E) Quantification of motor nerve branches in control (black), Ca_V_1.1^−/−^ (red), HSA-β-cat^−/−^ (green), and HSA-β-cat^−/−^; Ca_V_1.1^−/−^ (blue) mice using Sholl analysis as in (D). N numbers (of diaphragms analyzed) are indicated in brackets, mean ± SEM.

Recently, we demonstrated that the muscle calcium channel Ca_V_1.1 coordinates several aspects of the presynaptic motor nerve differentiation by initiating activity-driven muscle calcium signaling (Kaplan and Flucher, 2019), independently from its role in AChR patterning (Kaplan et al., 2018). That study suggested that expression of at least one putative *trans*-synaptic molecule displayed by muscle to the nerve must be under the control of Ca_V_1.1. To examine whether any of the previously identified retrograde signals (Rafuse et al., 2000; Schwander et al., 2004; Fox et al., 2007; Wu et al., 2012a, 2015; Jaworski and Tessier-Lavigne, 2012; Yumoto et al., 2012) are regulated by Ca_V_1.1, we performed qRT-PCR experiments using preparations of diaphragm muscles from Ca_V_1.1^−/−^ mice at E18.5. Compared to control mice, Fgf10, Slit2, Mmp9, and Integrin β1 were overexpressed in Ca_V_1.1^−/−^ mice (Figure 1A). Gdf2 (also known as Bmp9) expression showed a similar trend but this was not significantly different from control (p = 0.13, unpaired t-test). Overall, these data captured our attention because Slit2, Mmp9, Integrin β1, and Gdf2 (Bmp9) previously had been shown to be downregulated in P0 muscle-specific β-catenin knockout mice (Wu et al., 2015). This suggested that the lack of β-catenin or Ca_V_1.1 oppositely impacted transcription of several molecules important for NMJ formation.

The following lines of evidence led to the hypothesis that β-catenin and Ca_V_1.1 in muscle may have antagonistic functions in regulating motor nerve branching. First, muscle-specific β-catenin loss-of-function mutation leads to fewer secondary nerve branches (Li et al., 2008), whereas gain-of-function mutations increase the complexity of the arborization (Wu et al., 2012b; Liu et al., 2012). Secondly, lack of Ca_V_1.1 function causes hyper-branching of the motor nerve (Powell et al., 1984; Chen et al., 2011; Kaplan et al., 2018). Thirdly, β-catenin and Ca_V_1.1 have opposite effects on the expression of several identified retrograde signaling molecules (Figure 1A). Therefore, if β-catenin signaling and Ca_V_1.1 signaling converge on, and oppositely regulate common downstream regulators of presynaptic development (e.g., expression of Slit2 and/or other putative unidentified retrograde signals), then the concurrent loss of Ca_V_1.1 and β-catenin is expected to alleviate the presynaptic defects. In order to examine this possibility, we cross-bred HSA-cre, β-cat^flox/flox^, and Ca_V_1.1^+/−^ mice to generate muscle-specific β-catenin knockout mice in the Ca_V_1.1^−/−^ background (Figure 1B).

As hypothesized, motor nerve branching in E14.5 right diaphragms of HSA-β-cat^−/−^; Ca_V_1.1^−/−^ mice was clearly less than that in Ca_V_1.1^−/−^ mice, but more than in HSA-β-cat^−/−^ diaphragms, indicative of opposite regulatory actions of β-catenin and Ca_V_1.1 in this process (Figure 1C). Sholl analysis (Sholl, 1953) corroborated the qualitative assessment of our tissue preparations. For this we adapted the procedure using the primary branching point of the nerve after entering the muscle as a center (Figures 1D and 1E). Notably, we found that both in control and HSA-β-cat^−/−^ mice, nerve branching was restricted to the muscle center near the endplate zone with a substantially decreased number of branching points in HSA-β-cat^−/−^ mice compared to control mice. Whereas in HSA-β-cat^−/−^; Ca_V_1.1^−/−^ double-knockout mice, secondary branching occurred throughout the diaphragm similar to Ca_V_1.1^−/−^ mice, but with a substantially decreased number of branching points compared to Ca_V_1.1^−/−^ mice (Figure 1C, bottom row). Therefore, the absence of β-catenin leads to a decrease in nerve branching in both control and in Ca_V_1.1^−/−^ backgrounds. Conversely, the absence of Ca_V_1.1 leads to increased nerve branching throughout the diaphragm in both control and in HSA-β-cat^−/−^ backgrounds. These results indicate that the function of Ca_V_1.1 is to confine the retrograde mechanisms, which in turn confine motor nerve branching, to the central muscle territory; whereas the function of β-catenin is to induce sufficient nerve branching during early NMJ formation. Together, these observations suggest that β-catenin and Ca_V_1.1 employ complementary functions with opposite effects on the motor axons to promote nerve branching specifically in the center of the muscle fibers, and thus coordinate the innervation pattern of ingrowing motor nerves during the initial steps of NMJ development.

To test whether the roles of Ca_V_1.1 and β-catenin in the retrograde regulation of presynaptic development and the rescue of hyper-branching defects in Ca_V_1.1^−/−^ mice by knockout of β-catenin depended on Fgf10, Slit2, Mmp9, and/or Integrin β1 expression at E14.5, we performed qRT-PCR experiments on RNAs isolated from diaphragms. This analysis did not reveal any changes in mRNA expression levels of Fgf10, Slit2, Mmp9, and Integrin β1 in single and double knockouts (Figure S1), indicating that Ca_V_1.1 and β-catenin do not regulate nerve branching through transcriptional regulation of Fgf10, Slit2, Mmp9, and Integrin β1 at E14.5.

### Ca_V_1.1 and β-catenin cooperate to regulate AChR clustering in E14.5 diaphragms

**Figure 2:**
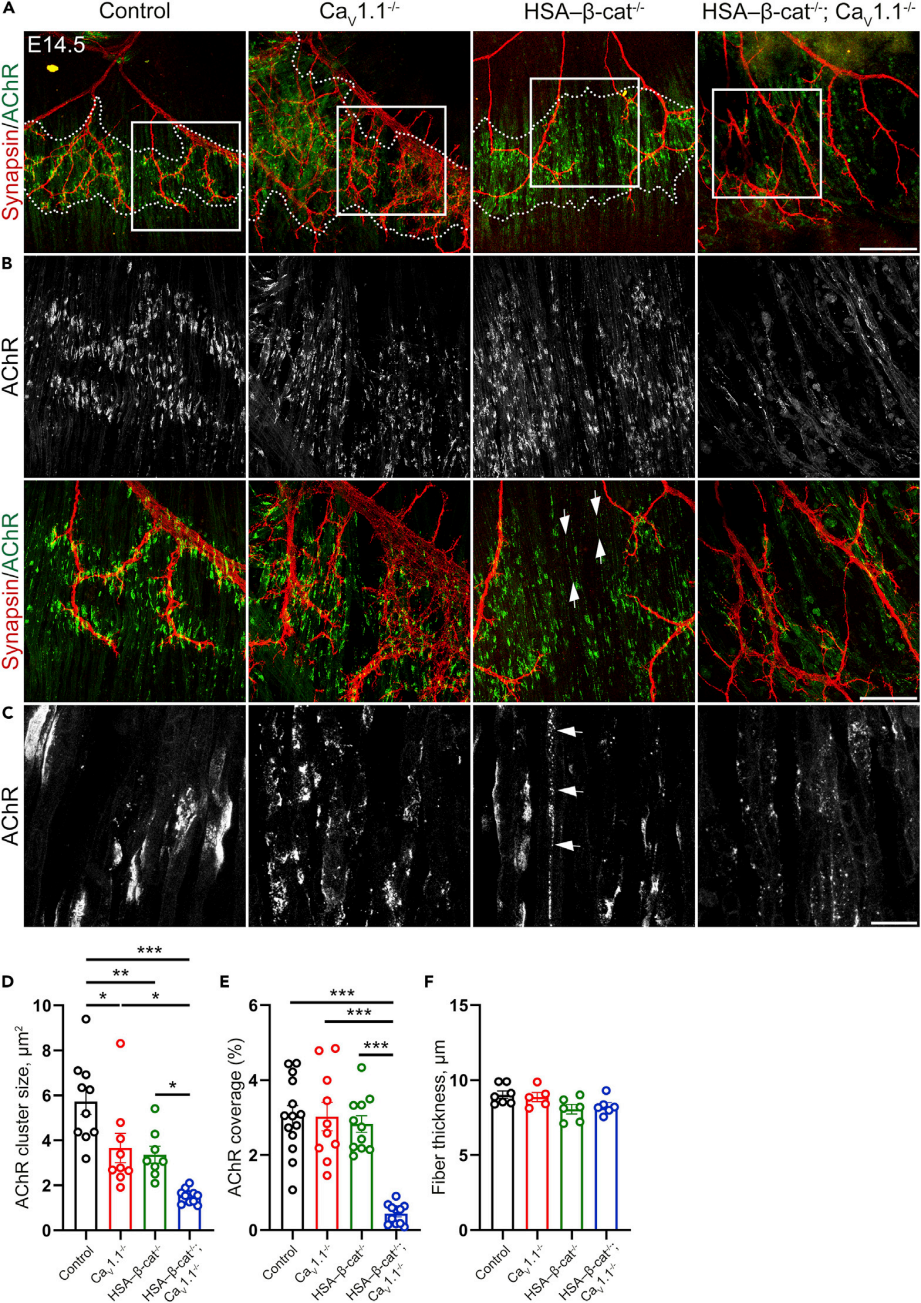
Cooperative functions of Ca_V_1.1 and β-catenin in regulation of AChR clustering in E14.5 diaphragms (A) Mouse whole-mount right diaphragm preparations stained with anti-synapsin (red) and α-BTX (green) at E14.5 to label motor nerve branches and AChR clusters, respectively, in control, Ca_V_1.1^−/−^, HSA-β-cat^−/−^, and HSA-β-cat^−/−^; Ca_V_1.1^−/−^ mice. The borders of the AChR cluster band are indicated with white dashed lines. (B) Framed areas in (A) demonstrating the stepwise deterioration of AChR clustering in control, single-, and double-knockout diaphragms, respectively, captured with a higher magnification objective. (C) Further 4× magnification showing α-BTX staining. HSA-β-cat^−/−^; Ca_V_1.1^−/−^ mice display severely disrupted AChR clustering. Arrows show some of the AChR clusters with a *beads-on-a-string*-like morphology in HSA-β-cat^−/−^ mice in (B and C). Scale bars, 200 μm for (A), 100 μm for (B), and 20 μm for (C). (D–F) Quantification of AChR cluster size (D), AChR coverage in muscle fibers at the endplate region (E), and muscle fiber thickness (F) in control, Ca_V_1.1^−/−^, HSA-β-cat ^−/−^, and HSA-β-cat^−/−^; Ca_V_1.1^−/−^ diaphragms. N ≥ 5 diaphragms from ≥5 litters; mean ± SEM; one way ANOVA: F_(3,34)_ = 15.11; p < 0.0001 for (D); ANOVA: F_(3,42)_ = 23.80; p < 0.0001 for (E); ANOVA: F_(3,20)_ = 3.021; p = 0.0538 for (F). Tukey's multiple comparison test: ∗p < 0.05, ∗∗p < 0.01, ∗∗∗p < 0.001, ^n.s^p > 0.05.

In addition to their retrograde actions in regulating nerve branching, both muscle β-catenin and Ca_V_1.1 were shown to regulate AChR clustering and patterning in E14.5 diaphragms. Muscle-specific β-catenin knockout mice displayed increased endplate bandwidth and AChR cluster size at E14.5 diaphragms (Li et al., 2008). The lack of Ca_V_1.1 expression also leads to increased endplate bandwidth at E14.5 diaphragms (Chen et al., 2011; Kaplan et al., 2018) (Figure 2A). We applied Alexa 488-BTX labeling of diaphragm muscles from E14.5 single and double knockouts to analyze postsynaptic AChR clustering during the initial phase of NMJ formation. Compared to control mice, diaphragms of both HSA-β-cat^−/−^ and Ca_V_1.1^−/−^ single-knockout mice showed a significantly decreased AChR cluster size (Figures 2B–2D). However, coverage of the muscle at the endplate band by AChR clusters was comparable in controls and single knockouts (Figure 2E). Moreover, in HSA-β-cat^−/−^ diaphragms, some of the fibers contained elongated and fragmented AChR clusters in a *beads-on-a-string*-like pattern (Figures 2B and 2C). This morphology may represent either a muscle-intrinsic, β-catenin-dependent developmental delay of AChR clustering or the effects of a decreased input from the motor nerve due to reduced innervation, resulting in deficient stabilization of synaptic clusters (agrin-dependent) and dispersal of non-synaptic clusters (ACh/activity-dependent).

Interestingly, in HSA-β-cat ^−/−^; Ca_V_1.1^−/−^ double-knockout mice, clustering of AChR was drastically disrupted in that most of the AChR staining appeared in diffuse aggregates of small sizes rather than forming compact AChR clusters (Figures 2A–2D). This was accompanied by substantially reduced AChR area covering muscle fibers in mice lacking both muscle β-catenin and Ca_V_1.1 (Figure 2E). Although some of the muscle fibers appeared disorganized and some regions in diaphragm were even devoid of muscle fibers, diaphragms of double knockouts contained long muscle fibers with sizes comparable to fibers in the single knockouts and controls (Figure 2F), indicating that the observed defects in clustering and membrane expression of AChRs are not secondary to deficient individual muscle fiber development. Overall, the additive effects observed in the double-knockout mice indicate that β-catenin and Ca_V_1.1 act in parallel to cooperatively regulate AChR clustering. Conversely, these data also suggest that the counteractive actions of β-catenin and Ca_V_1.1 in presynaptic development described above are not secondary effects of their cooperative functions in postsynaptic development.

### Perturbed NMJ and muscle fiber development in diaphragms of HSA-β-cat^−/−^; Ca_V_1.1^−/−^ mice at E18.5

**Figure 3:**
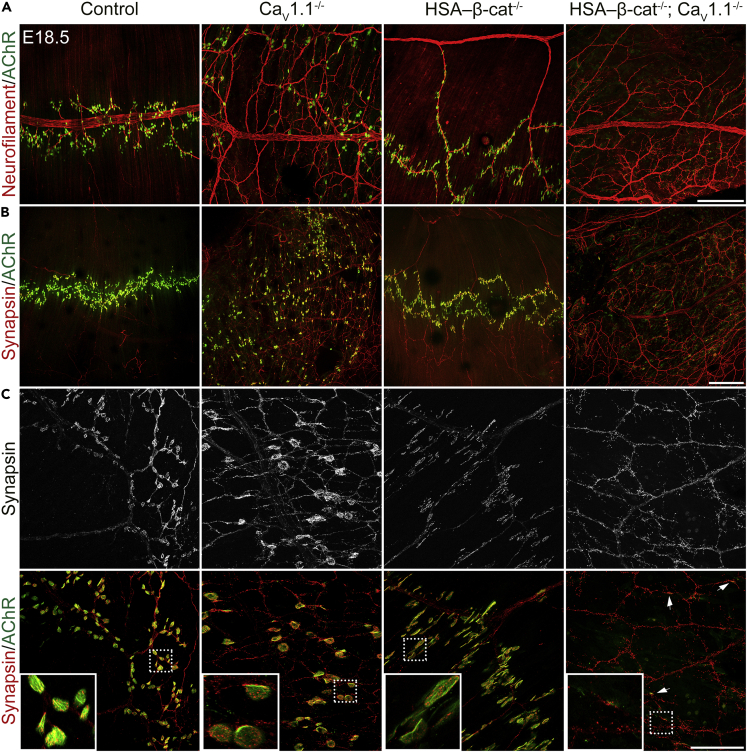
Disrupted NMJ formation in HSA-β-cat^−/−^; Ca_V_1.1^−/−^ diaphragms at E18.5 (A–C) Mouse whole-mount E18.5 diaphragms were stained with α-BTX (green) and neurofilament (A) or synapsin (B, C) antibodies (red) to label AChR clusters and motor nerve branches and nerve terminals, respectively. In HSA-β-cat^−/−^; Ca_V_1.1^−/−^ double-knockout diaphragms, motor nerve branching pattern displays similarity to that in Ca_V_1.1^−/−^ diaphragms; however, AChR clustering and synapsin accumulation at the nerve terminals are almost absent. Arrows show rare examples of rudimentary endplates opposite to tiny AChR clusters (C). Scale bars, 200 μm for (A), 300 μm for (B) and 100 μm for (C); insets show 3.5× magnifications of the AChR/synapsin clusters framed in (C).

The formation of the NMJ is subject to developmental regulation, and both β-catenin and Ca_V_1.1 play distinct roles in this process (Li et al., 2008; Kaplan et al., 2018). Principally, early defects in pre- or postsynaptic differentiation can become aggravated or alleviated as development progresses. In order to determine the effects of the combined loss of β-catenin and Ca_V_1.1 on NMJ formation at later developmental stages, we analyzed nerve branching, AChR clustering, and synapse formation in diaphragms of E18.5 mouse embryos. While in Ca_V_1.1-deficient mice, nerve branches covered even wider regions of the diaphragm than at E14.5, in HSA-β-cat^−/−^ embryos, the main nerve trunk was displaced to the inner edge of the diaphragm muscle and it projected strikingly fewer and longer axons to innervated AChR clusters, as shown previously (Li et al., 2008; Kaplan et al., 2018) (Figure 3A). These phenotypes in single knockouts were consistently observed in all mice examined in the course of this study. In stark contrast to the situation observed in E14.5 embryos, in E18.5 double knockouts, nerve branching was excessive and very similar to that in Ca_V_1.1-deficient mice. We analyzed three double-knockout mice, all of which showed a phenotype similar to that of Ca_V_1.1-deficient mice in that nerve covered nearly the entire diaphragm muscle. Also, different from the motor nerve phenotype of HSA-β-cat^−/−^ mice, in double-knockout diaphragms, the main nerve trunk was centrally located and projected long axons bilaterally, thus covering the entirety of the diaphragm (Figures 3A and 3B). This failure to improve the Ca_V_1.1^−/−^ phenotype in double-knockout mice indicates that at E18.5 knockout of β-catenin can no longer reverse the hyper-innervation defects observed in Ca_V_1.1-deficient mice. Apparently, at this later developmental stage, the action of Ca_V_1.1 on presynaptic growth dominates over the action of β-catenin in the retrograde regulation of innervation patterns. Probably, promotion of nerve branching by β-catenin-dependent retrograde signaling is active only in a narrow time window at the earlier stages of NMJ formation.

Strikingly, in HSA-β-cat^−/−^; Ca_V_1.1^−/−^ double-knockout mice, we rarely observed neuromuscular synapses. Although rare small AChR clusters formed, they looked very primitive compared to clusters in single knockouts or controls (Figure 3C). On the presynaptic side, the lacking accumulation of synapsin-positive vesicles indicated that nerve terminals rarely formed (Figures 3C and S2). Most likely, this is a consequence of the lacking postsynaptic differentiation. In fact, in E18.5 the concomitant knockout of Ca_V_1.1 and β-catenin resulted in a severely perturbed muscle fiber development, where small muscle fibers were scattered in a much disorganized manner (Figure S3). Together, with the normal size and partial alignment of muscle fibers at E14.5, this suggests that normal differentiation of diaphragm muscle after E14.5 critically depends on the activity of at least one of the two signaling pathways, Ca_V_1.1 or β-catenin. The concomitant failure of synapse formation in HSA-β-cat^−/−^; Ca_V_1.1^−/−^ double-knockout diaphragms suggests that the deficiency in presynaptic differentiation observed at E18.5 might be secondary to the defects in muscle development and AChR clustering.

### Differential effects of Ca_V_1.1 knockout on total β-catenin, active β-catenin, and TCF/Lef activity

**Figure 4:**
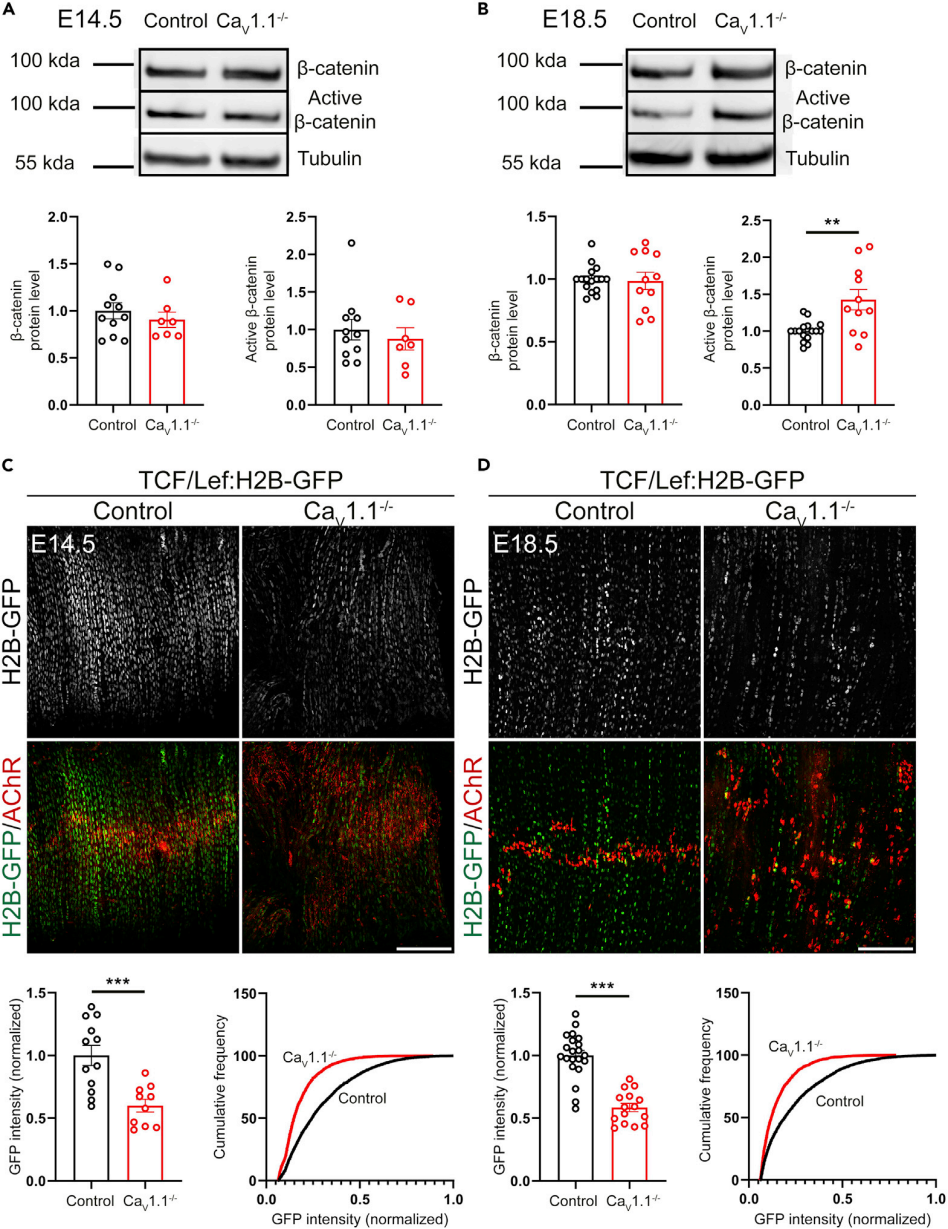
Regulation of TCF/Lef activity by Ca_V_1.1 (A and B) Western blot analysis of total and active β-catenin in Ca_V_1.1^−/−^ diaphragms at E14.5 (A) and E18.5 (B). N ≥ 7 diaphragms from 2 litters for (A) N ≥ 10 diaphragms from 4 litters for (B); mean ± SEM; t-test, ∗∗p = 0.0015 in (B). (C and D) Representative micrographs of diaphragms from TCF/Lef:H2B-GFP; Ca_V_1.1^+/?^ (control) and TCF/Lef:H2B-GFP; Ca_V_1.1^−/−^ mice labeled with Alexa 594-conjugated α-BTX (red) at E14.5 (C) and E18.5 (D) show decreased GFP fluorescence in Ca_V_1.1^−/−^ background at both developmental stages. Scale bars, 200 μm. Quantifications of GFP fluorescence intensity are provided in graphs below. Bar graphs show average fluorescence intensity from nuclei in each analyzed frame normalized to control mice from the same litter. Cumulative frequency graphs show the fluorscence intensity of nuclei analyzed normalized to the brightest nucleus from the same litter. Only unprocessed images were used for quantification. In the representative images, the brightness and contrast were adjusted to the same degree. N ≥ 2 litters, 4 mice, 10 frames, and 1708 nuclei for (C) and N ≥ 3 litters, three mice, nine frames, and 1483 nuclei for (D); mean ± SEM; t-test, ∗∗∗p = 0.0007 in (C), ∗∗∗p < 0.0001 in (D).

In order to explore whether Ca_V_1.1 regulates the expression or the activity of β-catenin, or both, we performed Western blot analysis with total β-catenin as wells as active β-catenin antibodies (Van Noort et al., 2002) on lysates of E14.5 and E18.5 diaphragms isolated from Ca_V_1.1^−/−^ mice and their control littermates. These experiments revealed no difference in protein levels of β-catenin at both E14.5 and E18.5. Although at E14.5, active β-catenin also was not different between control and Ca_V_1.1^−/−^ mice, at E18.5, a modest but statistically significant increase in expression of active β-catenin in Ca_V_1.1^−/−^ mice was detected (Figures 4A and 4B). These data indicate that Ca_V_1.1 activity does not directly regulate β-catenin protein expression at both E14.5 and E18.5, but it downregulates the activity of β-catenin during late fetal development.

In the canonical Wnt signaling pathway, β-catenin primarily regulates TCF/Lef (T-cell factor/lymphoid enhancer factor)-dependent transcription. TCF/Lef transcription factors then bind to Wnt response elements to regulate Wnt target genes (Niehrs, 2012; van Amerongen, 2012). To examine whether in embryonic mouse diaphragm muscle Ca_V_1.1 also regulates TCF/Lef-dependent transcription, we utilized a Wnt/β-cat reporter mouse line that has been widely used to analyze Wnt/β-catenin activity. Wnt/β-cat reporter mice carry a transgene including six copies of TCF/Lef-binding sites with *hsp68* minimal promoter and human histone protein H2B fused with eGFP (Ferrer-Vaquer et al., 2010). Because genotyping does not differentiate homozygous from hemizygous Wnt/β-cat reporter mice, we crossed Ca_V_1.1^+/−^ (GFP negative) mice with Ca_V_1.1^+/−^; TCF/Lef:H2B-eGFP (GFP positive) mice to ensure that GFP-expressing mice are hemizygous containing only one allele of TCF/Lef:H2B-eGFP transgene. If in mouse diaphragm β-catenin acted through TCF/Lef, we would expect an increased reporter gene activity in Ca_V_1.1^−/−^; TCF/Lef:H2B-eGFP mice at E18.5.

Surprisingly, in contrast to the unchanged total β-catenin protein levels and the increased active β-catenin in Ca_V_1.1^−/−^ mice, average GFP fluorescent intensity of nuclei in Ca_V_1.1^−/−^; TCF/Lef:H2B-eGFP was significantly reduced compared to that in Ca_V_1.1^+/?^; TCF/Lef:H2B-eGFP controls at both E14.5 and E18.5. At both time points, the cumulative GFP intensity plots of nuclei in Ca_V_1.1^−/−^; TCF/Lef:H2B-eGFP mice showed a left-shift indicating an overall decline in TCF/Lef activity in the absence of Ca_V_1.1 function (Figures 4C and 4D). These data indicate compromised TCF/Lef-dependent transcription in the absence of Ca_V_1.1, suggestive of a direct upregulation of TCF/Lef signaling by Ca_V_1.1 activity.

### Increased YAP activity in Ca_V_1.1^−/−^ mice at E18.5

**Figure 5:**
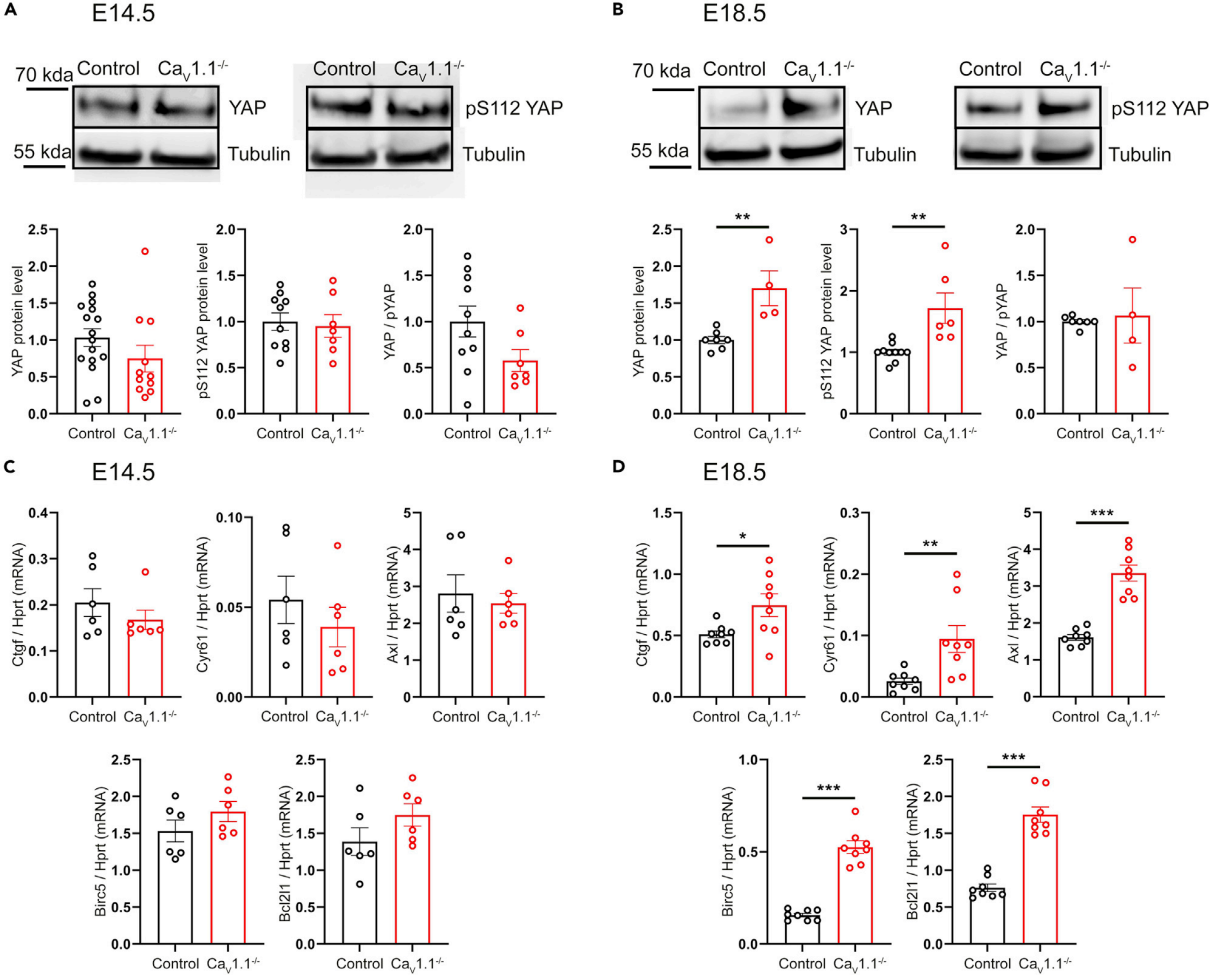
Increased YAP activity in the absence of Ca_V_1.1 (A and B) Western blot analysis of total YAP and YAP^pS^^112^ in Ca_V_1.1^−/−^ diaphragms at E14.5 (A) and E18.5 (B). N ≥ 7 diaphragms from 2 litters for (A) N ≥ 4 diaphragms from 4 litters for (B). mean ± SEM; t-test, ∗∗p = 0.004 for YAP, ∗∗p = 0.0025 for pYAP in (B). (C and D) qRT-PCR analysis of mRNAs of YAP target genes from diaphragm lysates of Ca_V_1.1^−/−^ and their control littermates at E14.5 (C) and E18.5 (D) shows increased expression levels of YAP target genes in the absence of Ca_V_1.1 at E18.5. N = 6 diaphragms from 6 litters for (C) and N = 8 diaphragms from ≥7 litters for (D). mean ± SEM; t-test, ∗p = 0.0272, ∗∗p = 0.0085, ∗∗∗p < 0.0001.

Recent studies indicate the existence of TCF/Lef-independent pathways of transcriptional regulation by β-catenin (Doumpas et al., 2019). The Hippo pathway effector YAP (Yes-associated protein) was shown to be an important transcriptional partner of β-catenin in cancerogenesis (Rosenbluh et al., 2012) and, more relevantly, in the control of NMJ formation and regeneration (Zhao et al., 2017). In HSA-YAP^−/−^ mice, similar to HSA-β-cat^−/−^ mice, secondary branching is decreased at E15.5 and P0. This simplified phenotype of nerve branching in both HSA-YAP^−/−^ and HSA-β-cat^−/−^ mice suggests that both might be involved in the same pathway to control motor innervation pattern and that increased nerve branching in Ca_V_1.1^−/−^ mice might result from an upregulation of the YAP pathway. Therefore, we analyzed YAP activity in Ca_V_1.1^−/−^ mice. First, we performed Western blot analysis with phosphorylated pYAPS112 (cytoplasmic) and total YAP antibodies. In E14.5 Ca_V_1.1^−/−^ diaphragms, Western blot analysis showed no difference in expression of either pYAPS112 or total YAP. However, at E18.5, we observed a significant increase in both pYAPS112 and total YAP protein expression (Figures 5A and 5B). The YAP/pYAP112 ratio was constant in Ca_V_1.1^−/−^ diaphragms at both E14.5 and E18.5. These data indicate a negative regulation of YAP expression by Ca_V_1.1 at later developmental stages. This parallels the increase of active β-catenin in Ca_V_1.1 knockout mice (Figure 4B) and thus suggests a possible regulation of the β-catenin/YAP transcriptional machinery by Ca_V_1.1-dependent calcium signaling.

In order to test the β-catenin-dependent and -independent transcriptional activity of YAP, we performed qRT-PCR analysis of TEAD/YAP target genes (*Ctgf*, *Cyr61*, and *Axl*) (Zhao et al., 2008; Xu et al., 2011; Zhang et al., 2011) and of β-catenin/YAP/TBX5 target genes (*Birc5* and *Bcl2l1*) (Rosenbluh et al., 2012) in Ca_V_1.1^−/−^ diaphragms. Again, the lack of Ca_V_1.1 did not lead to a change in mRNA levels of the analyzed genes at E14.5 diaphragms. However, at E18.5, we found an increase in expression of mRNAs of both TEAD and TBX5 target genes in Ca_V_1.1^−/−^ mice (Figures 5C and 5D). Consistent with the Western blot experiments, these results show that in late fetal development, Ca_V_1.1 regulates transcriptional activity of YAP. Interestingly, it does so by both the β-catenin-dependent (TBX5) and β-catenin-independent (TEAD) pathways, suggesting that YAP itself is the main target of Ca_V_1.1.

## Discussion

**Figure 6:**
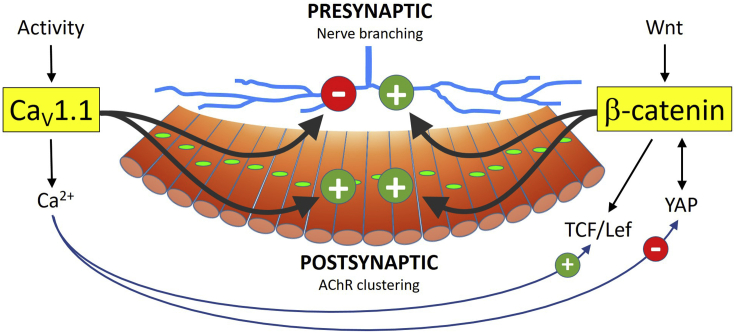
A possible model for coordinated regulation of NMJ formation by Ca_V_1.1 and β-catenin in mouse diaphragm muscle While Ca_V_1.1 acts downstream of electrical activity to restrict nerve branching to the endplate band, β-catenin (presumably downstream of Wnt) promotes sufficient nerve branching within this domain. Thus, their opposite but complementary roles establish the proper innervation pattern. On the other hand, postsynaptic AChR clustering and NMJ formation require the synergistic functions of Ca_V_1.1 and β-catenin, as AChR clustering and NMJ formation can be attained by either Ca_V_1.1 or β-catenin, but not in the absence of both of them. Ca_V_1.1 does not directly regulate β-catenin, but differentially regulates its transcriptional co-activators TCF/Lef and YAP, which might account for their differential roles in pre- and postsynaptic development.

The results of the current study demonstrate that Ca_V_1.1 and β-catenin work together in various ways in the regulation of distinct features of neuromuscular synaptogenesis and that Ca_V_1.1 differentially impacts the activity of transcriptional co-activators of β-catenin. Therefore, these findings reveal a crosstalk between two of the key regulatory mechanisms of NMJ formation and identify novel transcriptional targets of Ca_V_1.1, likely involved in activity-dependent calcium signaling in muscle (Figure 6).

The NMJ phenotypes displayed in the diaphragms of embryonic mice lacking either Ca_V_1.1 or β-catenin or both, establish opposite but complementary roles of Ca_V_1.1 and β-catenin in determining motor innervation pattern at the initial phase of NMJ development. Because Ca_V_1.1 is exclusively expressed in skeletal muscle, NMJ defects in DHPR-deficient mice can be rescued by specifically reintroducing the missing calcium channel subunit into muscle (Chen et al., 2011), and because β-catenin here is knocked out specifically in muscle, a presynaptic function of these molecules can be excluded. Therefore, the long-sought activity-dependent retrograde signals, which regulate motor nerve branching and innervation, likely are β-catenin-dependent as well. The primary function of Ca_V_1.1-dependent calcium signaling in muscle during the initial steps of innervation is to define the territory to which the motor nerve should grow, where to start branching and ultimately form synaptic connections (Kaplan et al., 2018; Kaplan and Flucher, 2019). Accordingly, without Ca_V_1.1, in both Ca_V_1.1^−/−^ and HSA-β-cat^−/−^; Ca_V_1.1^−/−^ double-knockout mice, nerve branching occurs unrestricted all over the muscle. The primary role of β-catenin is to promote nerve branching. Without β-catenin, the number of branch points is reduced, irrespective of the additional presence or absence of Ca_V_1.1, in HSA-β-cat^−/−^ and HSA-β-cat^−/−^; Ca_V_1.1^−/−^ mice, respectively. Notably, the paucity of nerve branching in β-catenin knockout diaphragm is not caused by a reduction in AChR clusters in the postsynaptic membrane, because AChR cluster size and coverage are similar to that in Ca_V_1.1-deficient diaphragm. Together, the opposing actions of Ca_V_1.1 and β-catenin in muscle on the ingrowing motor nerve assure sufficient nerve branching exclusively in the central endplate band of the muscle.

Interestingly, the active time window for their complementary presynaptic action appears to be limited to early stages of NMJ formation. Later in development, β-catenin knockout no longer is capable of rescuing the hyper-innervation defects caused by Ca_V_1.1-deficiency. This suggests a mechanism by which in normal development, nerve branching is self-limited in that it ceases when the growth cones reach their targets, collapse, and differentiate functional nerve terminals. Promotion of branching by β-catenin assures rapid completion of this process. When this self-limiting process fails, because the postsynaptic target area is not defined in the double knockouts, branching will continue at the low rate persisting in β-catenin-deficient muscle and eventually also cover the entire diaphragm. Notably, this process, resulting in similar nerve branching phenotypes in Ca_V_1.1^−/−^ and in HSA-β-cat^−/−^; Ca_V_1.1^−/−^ mice, likely is the consequence of the severe postsynaptic defects in the double knockouts at E18.5. Because of the almost complete lack of AChR clusters (i.e. postsynaptic target structures), the growth cones will keep growing, allowing sufficient time to cover the entire diaphragm.

Together, these findings also highlight the distinct modes of action of Ca_V_1.1 and β-catenin in pre- and postsynaptic development. Whereas presynaptically Ca_V_1.1 and β-catenin exert opposing effects on nerve branching, postsynaptically they show synergistic effects on AChR clustering. Both individual knockouts cause widening of the endplate band and a reduction in AChR cluster size. In the double knockouts, this phenotype is exaggerated in that there is an extreme reduction of AChR cluster number and size, with no recognizable restriction of the remaining clusters within the diaphragm muscle. This demonstrates that each signaling mechanism, Ca_V_1.1-dependent and β-catenin-dependent, can maintain a certain degree of AChR clustering and patterning in the absence of the respective other. Thus, the two signaling mechanisms possess partially redundant actions on the formation and organization of AChR clusters. Previous cell culture studies showed the importance of Ca_V_1.1 and β-catenin for agrin-induced AChR clustering (Milholland et al., 2007; Zhang et al., 2007). This is consistent with our data at E14.5, where we see substantially reduced AChR cluster sizes in both single knockouts. However, this is no longer the case at E18.5, where AChR cluster size is normal or even increased (Figure S2, Li et al., 2008). Because at E18.5 agrin-mediated AChR clustering is expected to play a bigger role than at E14.5, the direct effects of Ca_V_1.1 and β-catenin on agrin-induced AChR clustering described *in vitro* cannot account for the regulation of AChR clustering and postsynaptic differentiation *in vivo*. During the coordinated differentiation of nerve and muscle in the developing diaphragm, additional factors seem to play a decisive role in the absence of Ca_V_1.1 or β-catenin.

It is generally accepted that pre- and postsynaptic development are highly interdependent. Therefore, an effect on one might indirectly influence the other. However, our findings showing that concomitant knockout of Ca_V_1.1 and β-catenin has rectifying effects on the presynaptic phenotype, while worsening effects on the postsynaptic phenotype at E14.5, strongly suggest that Ca_V_1.1 and β-catenin-mediated downstream signaling cascades diverge to differentially control the mechanisms regulating pre- and postsynaptic development. Although the signaling cascade downstream of muscle activity/Ca_V_1.1 is reasonably well understood (e.g. CaMKII, Cdk5, and MuSK) (Tang et al., 2004; Lin et al., 2005; Chen et al., 2007, 2011; Kaplan et al., 2018), little is known about downstream effects of β-catenin in skeletal muscle responsible for regulating postsynaptic differentiation. HSA-β-cat^−/−^ mice display mild aberrancies in the distribution and size of AChR clusters (Figure 2 and 3) (Li et al., 2008). However, hitherto it was not clear to what extent those defects are due to the decrease in innervation. In the present study, we show a direct role of β-catenin in regulating AChR clustering and formation of NMJs when innervation is rescued (E14.5) or even excessive (E18.5) in HSA-β-cat^−/−^; Ca_V_1.1^−/−^ double-knockout mice, thus strongly suggesting a direct involvement of β-catenin signaling in clustering and patterning of AChRs, independent of the degree of innervation.

Previously, it has been shown that the function of β-catenin as transcriptional regulator is required for proper NMJ formation *in vivo* (Wu et al., 2015). When testing whether β-catenin-dependent transcriptional activity was miss-regulated in the absence of Ca_V_1.1-mediated calcium signaling, we found that TCF/Lef-dependent transcription is promoted, whereas YAP-dependent transcription is suppressed by Ca_V_1.1. Thus, these observations identify hitherto unnoticed pathways by which activity might control transcription in skeletal muscle. The concomitant upregulation of active β-catenin and YAP, as well as β-catenin-dependent YAP target genes in Ca_V_1.1-deficient mice strongly supports the notion that activity and Ca_V_1.1-dependent calcium signaling converges with this non-conventional β-catenin pathway. The parallel upregulation of β-catenin-independent YAP target genes suggests that YAP itself is the point of action for the Ca_V_1.1 pathway. Interestingly, the retrograde signals downstream of β-catenin, Slit2, Fgf10, and Mmp9 are downregulated by Ca_V_1.1 at E18.5 and have also been associated with YAP-dependent transcription (Lavado et al., 2014; Pan et al., 2017; Volckaert et al., 2017; Zhao et al., 2017). The increased expression of both active β-catenin and YAP at E18.5, but not at E14.5, might also explain the differential expression patterns of Slit2, Fgf10, and Mmp9 in Ca_V_1.1-deficient mice at these two developmental stages.

Because TCF/Lefs are transcriptional partners of β-catenin and their activity is compromised in the absence of Ca_V_1.1, whereas β-catenin expression is unaltered, it is likely that Ca_V_1.1 controls TCF/Lef activity independently of β-catenin. Therefore, signal transduction between Ca_V_1.1 and TCF/Lef should act downstream or parallel to β-catenin. Several molecules were identified having agonistic or antagonistic effects on the β-catenin-TCF/Lef pathway, including RNF14 (Wu et al., 2013), PARP-1 (Idogawa et al., 2005), TBL1 (Li and Wang, 2008), ICAT (Tago et al., 2000), Chibby (Takemaru et al., 2003), and Groucho/TLE (Roose et al., 1998). It will be interesting to test whether Ca_V_1.1 activity regulates TCF/Lef-dependent transcription through any of these molecules. In fact, a recent study reported that nuclear YAP interacts with Groucho/TLE transcriptional co-repressors to block TCF/Lef-dependent transcription (Li et al., 2020). Moreover, cytoplasmic YAP phosphorylated at Ser127 (Ser112) by the Hippo pathway suppresses nuclear translocation of β-catenin, leading to decrease in TCF-dependent transcriptional activity of β-catenin (Imajo et al., 2012). Therefore, both nuclear and cytoplasmic YAP appear to have suppressive roles in TCF/Lef activity. Consistent with this, we observed an increase in expression and Ser112 phosphorylation of YAP and decrease in TCF/Lef reporter activity in Ca_V_1.1-deficient mice. However, how Ca_V_1.1 exactly controls the activities of TCF/Lef and YAP, and whether or not these regulatory pathways are important for NMJ development, remains to be further investigated.

Activation of β-catenin-independent YAP target genes further suggests an involvement of the Hippo pathway in activity-dependent regulation of NMJ formation. Because the Hippo pathway, which controls the phosphorylation and activity of YAP, is regulated by mechanical cues (Rausch and Hansen, 2020), and muscles of Ca_V_1.1-deficient mice fail to contract (Tanabe et al., 1988), this contractile inactivity may explain the aberrant activation of YAP independently of β-catenin. A recent study reporting an increase in pYAP112 and total YAP levels in denervated muscle (Watt et al., 2015) is consistent with this notion. Interestingly, upregulation of the YAP pathway is detected only at E18.5 but not at E14.5. Because contractile activity is increased during development, at E14.5, spontaneous contraction may not be sufficient to downregulate YAP; therefore, the lack of calcium signaling in Ca_V_1.1-null mice does not affect YAP signaling at this early developmental stage. However, at E18.5, contractile activity may be sufficiently high to show downregulation of YAP signaling in normal relative to Ca_V_1.1-null muscles. On the other hand, because activity and Ca_V_1.1 control MuSK (Valenzuela et al., 1995; Tang and Goldman, 2006; Chen et al., 2011; Kaplan et al., 2018) and integrin β1 expression (Figure 1A), and because it has been recently shown that integrin-LRP4/MuSK pathways regulate YAP phosphorylation and activity through the Hippo pathway in liver cancer development (Chakraborty et al., 2017), it is possible that integrins and MuSK represent activity-dependent regulators of YAP signaling also in skeletal muscle.

In conclusion, this study offers a better understanding of how two key regulators of NMJ formation, Ca_V_1.1 and β-catenin, act together to build proper nerve-muscle synapses. Importantly, their interconnected functions depend on whether they impact pre- or postsynaptic processes, and whether they act during early or late NMJ development; thus highlighting the complexity of their interactions in NMJ formation. One possible connection between these two signaling pathways is that Ca_V_1.1 controls β-catenin-mediated transcription via canonical and non-canonical β-catenin signaling. Together, these findings provide a conceptual framework describing how NMJ formation is controlled jointly by Ca_V_1.1 and β-catenin.

### Limitations of the study

Firstly, it must be noted that the phenotypes displayed by the motor nerve, AChR clusters, and muscle fibers presented here are derived from costal diaphragm muscle, which, because of its superior accessibility for microscopic analysis and the stereotypical innervation patterns, represents the prime model system for studying NMJ formation in mammals. In muscles of different ontogeny and morphology, the effects of Ca_V_1.1 and β-catenin knockout may be less clear. For example, we noted that in crural diaphragm muscle nerve branching displays little defects, if any, in the HSA-β-cat^−/−^ mice (Li et al., 2008). Such heterogeneity adds further complexity to the regulatory mechanisms and must be considered when interpreting the results, as for instance in whole diaphragm preparations (i.e. protein lysates, RNA preps), changes of specific signaling proteins might be diluted or masked by the content of non-affected tissue.

Secondly, this study reveals that Ca_V_1.1 and β-catenin differentially cooperate in the regulation of pre- and postsynaptic development of the NMJ and that the interaction of these two pathways changes during fetal development. We identified two transcriptional partners of β-catenin regulated by Ca_V_1.1, TCF/Lef and YAP. However, the exact unifying mechanism downstream of Ca_V_1.1 and β-catenin for NMJ formation remains unclear. Further studies are needed to conclusively demonstrate the importance of these transcriptional regulations for NMJ formation downstream of Ca_V_1.1. To this end, NMJ formation should be analyzed in genetic mouse models combining Ca_V_1.1 deficiency with altered expressions of TCF/Lef and YAP.

## STAR★Methods

### Key resources table

**Table:** 

REAGENT or RESOURCE	SOURCE	IDENTIFIER
**Antibodies**		

Rabbit polyclonal anti-Synapsin1/2	Synaptic Systems	Cat# 106 002, RRID:AB_887804
rbMuSK	Dr. Markus A. Rüegg	194T/Nsk-2
Rabbit polyclonal anti-Neurofilament 200	Sigma-Aldrich	Cat# N4142, RRID:AB_477272
Mouse monoclonal anti-β-catenin	Cell Signaling Technology	Cat# 2698, RRID:AB_1030945
Mouse monoclonal anti-active β-catenin	Millipore	Cat# 05-665, RRID:AB_309887
Mouse monoclonal anti-Yap	Santa Cruz Biotechnology	Cat# sc-101199, RRID:AB_1131430
Rabbit monoclonal anti-Phospho-Yap (Ser127)	Cell Signaling Technology	Cat# 13008, RRID:AB_2650553
Mouse alpha Tubulin	Abcam	Cat# ab7291, RRID:AB_2241126

**Chemicals, peptides, and recombinant proteins**		

α-Bungarotoxin Alexa488-conjugate	Invitrogen	Cat# B13422
α-Bungarotoxin Alexa594-conjugate	Invitrogen	Cat# B13423

**Critical commercial assays**		

RNeasy Fibrous Tissue Mini Kit	Qiagen	Cat# 74704
SuperScript II Reverse Transcriptase	Invirtogen	Cat# 100004925
TaqMan Universal Master Mix	Applied Biosystems	Cat# 4440040

**Experimental models: Organisms/strains**		

Mouse: Dysgenic (Ca_V_1.1^+/-^)	Tanabe et al., 1988	NA
Mouse: B6.129-Ctnnb1tmKem/KnwJ	The Jackson Laboratory	JAX: 004152
Mouse: B6.Ct-Tg(ACTA1-cre)79Jme/J	The Jackson Laboratory	JAX:006149
Mouse: Tg(TCF/Lef1-HIST1H2BB/EGFP)61Hadj/J	The Jackson Laboratory	JAX:013752

**Oligonucleotides**		

Ca_V_1.1 Primer Forward: GCT TTG CAG ATG TTC GGG AAG ATC GCC ATG	Eurofins	NA
Ca_V_1.1 Primer Reverse: GCA GCT TTC CAC TCA GGA GGG ATC CAG TGT	Eurofins	NA
β-cat(floxed) Primer Forward: AAG GTA GAG TGA TGA AAG TTG TT	Eurofins	NA
β-cat(floxed) Primer Reverse: CAC CAT GTC CTC TGT CTA TTC	Eurofins	NA
HSA-cre Primer Forward: GCG GTC TGG CAG TAA AAA CTA TC	Eurofins	NA
HSA-cre Primer Reverse: GTG AAA CAG CAT TGC TGT CAC TT	Eurofins	NA
Internal Positive Control Primer Forward: CTA GGC CAC AGA ATT GAA AGA TCT	Eurofins	NA
Internal Positive Control Primer Reverse: GTA GGT GGA AAT TCT AGC ATC ATC C	Eurofins	NA
TCF/Lef:GFP Primer Forward: ACA ACA AGC GCT CGA CCA TCA C	Eurofins	NA
TCF/Lef:GFP Primer Reverse: AGT CGA TGC CCT TCA GCT CGA T	Eurofins	NA

**Software and algorithms**		

Image Studio Lite 5.0	LI-COR	https://www.licor.com/bio/image-studio-lite/ (Discontinued)
Las AF 2.6.3	Leica Microsystems CMS GmbH	https://www.leica-microsystems.com/
Zen 3.2 (blue edition)	Carl Zeiss Microscopy GmbH	https://www.zeiss.com/
Design & Analysis Software 2.4.3	Thermo Fisher Scientific	https://www.thermofisher.com/at/en/home/global/forms/life-science/quantstudio-6-7-pro-software.html
GraphPad Prism 9	GraphPad	https://www.graphpad.com/
Adobe Photoshop CS6 13.0.1	Adobe Systems Incorporated	https://www.adobe.com/
MetaMorph 7.8.0.0	Molecular Devices	https://www.moleculardevices.com/

### Resource availability

#### Lead contact

Further information and requests for resources and reagents should be directed to and will be fulfilled by the lead contact, Mehmet M. Kaplan (mehmet.kaplan@i-med.ac.at).

#### Materials availability

This study did not generate new unique reagents.

### Experimental model and subject details

#### Mice

All animal protocols conformed to the guidelines of the European Community (86/609/EEC) and were approved by the Austrian Ministry of Science (GZ: 2020-0.073.957 and GZ: 2020-0.073.961). Ca_v_1.1^-/-^ (Tanabe et al., 1988), HSA–β-cat^−/−^ (Li et al., 2008), and TCF/Lef:H2B-eGFP (Ferrer-Vaquer et al., 2010) mice have been described previously. β-cat^floxed/floxed^; Ca_v_1.1^+/−^ mice were generated by crossing β-cat^floxed/floxed^ mice (purchased from Jaxson lab) and Ca_v_1.1^+/−^ mice. In parallel, HSA–Cre mice were crossed with β-cat^floxed/floxed^ mice to generate HSA–β-cat^floxed/+^ mice, which were then crossed with Ca_v_1.1^+/−^ mice to obtain HSA–β-cat^floxed/+^; Ca_v_1.1^+/−^ mice. β-cat^floxed/floxed^; Ca_v_1.1^+/−^ mice were crossed with HSA–β-cat^floxed/+^; Ca_v_1.1^+/−^ mice to obtain HSA–β-cat^floxed/floxed^; Ca_v_1.1^+/−^ (HSA–β-cat^−/−^; Ca_v_1.1^−/−^) embryos. At both E14.5 and E18.5, the observed number of HSA–β-cat^−/−^, Ca_v_1.1^−/−^ and HSA–β-cat^−/−^; Ca_v_1.1^−/−^ embryos collected, genotyped and analyzed during the course of this study corresponded to the expected ratio of Mendelian inheritance. Embryos heterozygous for HSA–β-cat and/or Ca_v_1.1 did not show any phenotype. Control embryos analyzed were carrying at least one Ca_v_1.1 and one β-catenin expressing allele. Ca_v_1.1^+/−^ mice were crossed with TCF/Lef:H2B-eGFP mice (purchased from Jaxson lab) to generate Ca_v_1.1^+/−^; TCF/Lef:H2B-eGFP mice, which were then crossed with Ca_v_1.1^+/−^ mice to obtain Ca_v_1.1^−/−^; TCF/Lef:H2B-eGFP embryos. Either Ca_v_1.1^+/+^; TCF/Lef:H2B-eGFP or Ca_v_1.1^+/−^; TCF/Lef:H2B-eGFP mice were used as controls. During mating periods sperm plugs were checked daily at 8:00 a.m. The day on which a sperm plug was detected was counted as embryonic day E0.5. Embryos were collected at the indicated days of pregnancy by cesarean section of sacrificed pregnant mice.

### Method details

#### Immunohistochemistry and image processing

*For whole-mount diaphragms:* The upper trunk of the mouse embryos containing the ribcage, liver, diaphragm and lungs was fixed by immersion in 4% paraformaldehyde in 0.1 M phosphate buffer (pH 7.2) for 1 h at room temperature. Diaphragms were dissected in PBS and incubated in 0.1 M glycine in PBS for 1 h at room temperature, permeabilized and blocked in PBS containing 1% BSA, 5% normal goat serum (NGS) and 0.2% Triton X-100 overnight at 4 °C. Primary antibodies for rbSynapsin (1/10,000, Synaptic System), rbNeurofilament (1/1,000, Sigma-Aldrich) or rbMuSK (194 T [Nsk-2]; 1/2,000) were applied at 4°C overnight. Then muscle samples were washed three times at 1 h intervals and incubated with anti-rabbit Alexa594 (1/4,000), anti-rabbit Alexa488 (1/4,000), anti-mouse Alexa594 (1/4,000) according to the primary antibodies applied and/or α-BTX conjugated with Alexa488 or Alexa594 (1/8,000, all from Invitrogen) for 2 h at room temperature. After extensive washing, diaphragms were mounted in Vectashield. Images were captured on a Leica microsystems SP5 laser scanning confocal microscopy using LasAF acquisition software (Leica microsystems). Fluorescence was excited using the 488 nm and 561 nm laser lines and recorded at a bandwidth of 493-556 nm (green channel) and 566-752 nm (red channel), respectively. 8-bit images with 1024x1024 pixels were acquired at 400 Hz scan speed. Maximum projections of acquired z-serial images were assembled and analyzed with Metamorph software. Micrographs in Figure 3C were obtained by a Zeiss LSM980 Airyscan microscope with a 25X objective. Image processing and assembly of the figures was performed using Adobe Photoshop CS6. Only linear adjustment was applied to correct brightness and contrast.

#### Quantitative Real Time-PCR

Total RNAs were extracted from diaphragm muscles at E14.5 or E18.5 using RNeasy Fibrous Tissue kit (Qiagen). cDNAs were synthesized by Superscripts II Reverse Transcriptase (Invirtogen). We performed RT-PCR by using TaqMan gene expression assays in master mix with QuantStudio 6 Pro RT-PCR system (Thermofisher Scientific, catalog #, A43180). We measured duplicates for each sample and used Hprt (Hypoxanthine guanine phosphoribosyl transferase) as a reference gene. For relative RT-PCR, the difference between C_t_ values of Hprt and gene of interest was used to calculate relative expression. Taqman gene expression assays were purchased from Applied Biosystems. (Assay IDs: Hprt, Mm00446968_m1; Fgf7, Mm00433291_m1; Fgf10, Mm00433275_m1; Gdf2, Mm00807340_m1; Integrin β1, Mm01253230_m1; Lrp4, Mm00554326_m1; Ncam, Mm01149710_m1; Mmp9, Mm00442991_m1; Slit2, Mm01216521_m1; Ctgf, Mm01192933_g1; Cyr61, Mm00487498_m1; Axl, Mm00437221_m1; Birc5, Mm00599749_m1; Bcl2l1, Mm00437783_m1)

#### Western Blot

E14.5 or E18.5 diaphragms were dissected in ice-cold PBS and scraped in RIPA buffer containing 50 mM TRIS-HCl (pH:8), 50 mM NaCl, 10 mM NaF, 0.5 mM EDTA, 10%SDS, 10% glycerol, 1%igepal, 1x protease inhibitor complete cocktail (Roch). Lysates were centrifuged at 13,100 g for 15 minutes at 4°C. A BCA assay (Thermo Scientific) was used to determine protein concentrations. We separated 10 μg of diaphragm lysates by 4–12% SDS-PAGE at 80V for 30 minutes followed by 160V for 90 minutes, transferred the protein to a PVDF membrane at 25V, 100 mA for 2,5-3 hours at 4°C using a semi-dry blot (VWR, 700-1220). The membrane was stained with mouse β-catenin (1/1,000, New England BioLabs, L87A12), mouse active β-catenin (1/1,000, Merck Millipore Clone 8 × 10^7^), mouse YAP (1/500, Santa Cruz Biotechnology, 63.7), rabbit pYAP127 (1/1,000, Cell Signaling Technology, D9W2I), or α-tubulin (1/2000, Abcam, DM1A) overnight at 4°C and then with secondary antibodies conjugated with HRP for 1 hour at room temperature. ECL Supersignal West Pico was used to detect the chemiluminescence, which was visualized with ImageQuant LAS4000. Image Studio Lite was used to quantify Western Blot bands. Values were first normalized to tubulin and then to the average of control mice for each litter.

### Quantification and statistical analysis

#### Sholl analysis

We adapted the Sholl analysis from (Sholl, 1953). Synapsin-labeled diaphragms at E14.5 were imaged using an SP5 laser scanning confocal microscope equipped with a 10× objective. With Metamorph software, we assigned the entry point of the right phrenic nerve into the right costal diaphragms as the center/origin for the analysis. Concentric circles were drawn with the radius increasing by 50 μm at every consecutive circle. The nerve branches crossing the circles were manually counted and plotted in a graph.

#### AChR cluster size

BTX-labeled diaphragms at E14.5 were imaged using an SP5 laser scanning confocal microscope equipped with a 63× objective. With Metamorph software, a maximal projection of a 15 μm thick stack was assembled. A threshold was applied to the image to include AChR clusters. Areas smaller than 0.5 μm^2^ were excluded. We used integrated morphometry analysis to extract the data from the analyzed areas. Each region was considered as an AChR cluster. The average size of regions in each image was plotted in a graph. For E18.5 AChR cluster size analysis, images were obtained with an SP5 laser scanning confocal microscope equipped with a 63X or 40× objectives. In Metamorph software, a maximal projection of a 20 μm thick stack was assembled and a threshold was applied. Areas covered by AChR clusters were measured by drawing and shrinking a circle. The size of each synapse was plotted in a graph.

#### AChR cluster area/muscle area

BTX-labeled diaphragms at E14.5 were imaged using an SP5 laser scanning confocal microscope equipped with a 63× objective. With Metamorph software, a 10 μm in z-volume thick area was assembled. First, a threshold was applied to the image to include AChR clusters. Integrated morphometry analysis extracted data from the analyzed areas was summed up to obtain the total AChR area in the image. Then, a second threshold applied to muscle fibers, taking advantage of muscle autofluorescence. The total AChR area was normalized to the total muscle area. Muscle fiber size was analyzed as described previously (Kaplan et al., 2018).

#### GFP intensity analysis in TCF-reporter mice

GFP fluorescence from diaphragm muscles was captured using an SP5 confocal microscopy with a 40× objective. With Metamorph software, a 15 μm in z-volume thick area was assembled and a threshold was applied to exclude nuclei-free regions. Integrated morphometry analysis extracted intensity values from each nucleus. Data from each image were averaged and plotted in a graph as GFP intensity for that image. Additionally, GFP intensity from all nuclei from each separate experiment were normalized to the brightest nucleus and plotted as a cumulative frequency graph using GraphPad. Because Ca_v_1.1^+/+^ and Ca_v_1.1^+/−^ mice show an identical phenotype, both genotypes were pooled and analyzed as controls and referred by TCF/Lef:H2B-GFP; Ca_V_1.1^+/?^ mice.

#### Statistical analysis

All statistical analyses were performed with GraphPad Prism Software 9. N numbers are indicated in the figure legends. Mean ± sem (standard error of the mean) was used to present all data. The two-tailed student t-test was applied to assess statistical differences between two genotypes. Ordinary one-way ANOVA was applied to assess statistical significance for the differences between more than two genotypes. A p < 0.05 ANOVA value was considered as significant, as indicated in the figure legends. ANOVA was followed by a Tukey’s multiple comparisons post hoc test to reveal statistically significant differences among experimental groups.

## Data Availability

This paper does not report original code. All data produced in this study are included in the published article and its supplemental information, or are available from the lead contact upon request.
