# Supplementary material for: Counteractive and cooperative actions of muscle β-catenin and CaV1.1 during early neuromuscular synapse formation
Source: iScience. 2022 Mar 4;25(4):104025. doi: 10.1016/j.isci.2022.104025 (PMC8941212; doi:10.1016/j.isci.2022.104025)
Supplement: Document S1. Figures S1–S3 [file mmc1.pdf]

iScience, Volume *25*

Supplemental information

Counteractive and cooperative actions of muscle β-catenin and $Ca_V1.1$ during early neuromuscular synapse formation

Mehmet Mahsum Kaplan and Bernhard E. Flucher

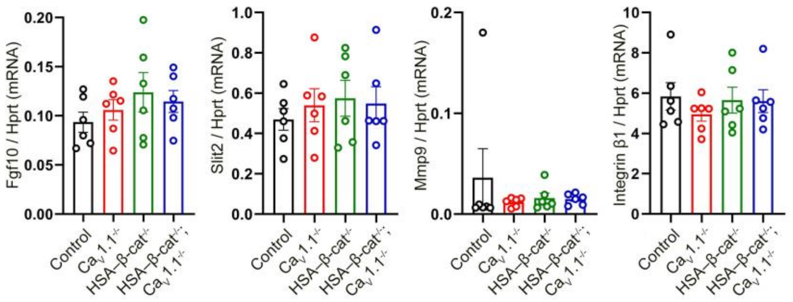

Supplemental Figure 1, related to Figure 1: $Ca_V1.1$ and β-catenin do not regulate Fgf10, Slit2, Mmp9 and Integrin β1 expression at E14.5. qRT-PCR analysis of mRNAs of Fgf10, Slit2, Mmp9 and Integrin β1 in control (black), $Ca_V1.1^{-/-}$ (red), HSA–β-cat$^{-/-}$ (green), and HSA–β-cat$^{-/-}$; $Ca_V1.1^{-/-}$ (blue) mice at E14.5 shows no statistically significant difference. N=6 diaphragms from 6 litters; mean ± sem; one way ANOVA: $F_{(3,20)}$=1.753; p = 0.1886 for Fgf10; ANOVA: $F_{(3,20)}$=0.6243; p = 0.6076 for Slit2; ANOVA: $F_{(3,20)}$=0.7336; p = 0.5442 for Mmp9; ANOVA: $F_{(3,20)}$=0.4248; p = 0.7373 for Integrin β1.

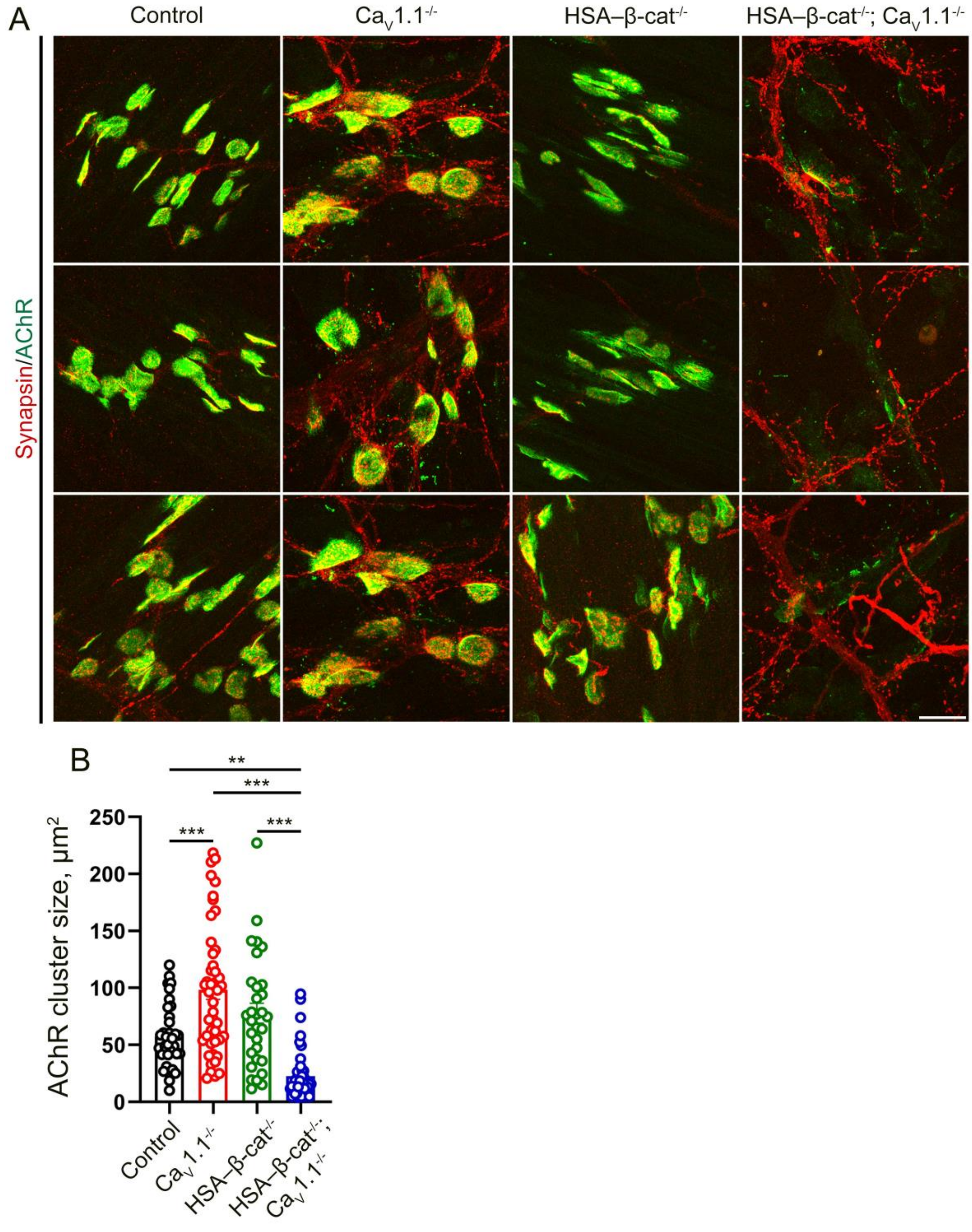

Supplemental Figure 2, related to Figure 3: Defective synapse formation in HSA–β-cat$^{-/-}$; Ca$_V$1.1$^{-/-}$ double-knockout mice. (A) Double labeling of synaptic vesicles and AChR clusters with synapsin antibody (red) and BTX (green) in E18.5 diaphragms of control, Ca$_V$1.1$^{-/-}$, HSA–β-cat$^{-/-}$, and HSA–β-cat$^{-/-}$; Ca$_V$1.1$^{-/-}$ mice reveals severely perturbed NMJs in the double-knockouts as compared to control and single knockouts. Scale bar, 20μm. **(B)** Quantification of AChR cluster size in control, Ca$_V$1.1$^{-/-}$, HSA–β-cat $^{-/-}$, and HSA–β-cat$^{-/-}$; Ca$_V$1.1$^{-/-}$ diaphragms at E18.5. N =2 diaphragms from 2 litters at least 36 synapses analyzed for each genotype; mean ± sem; one way ANOVA: $F_{(3,154)}$= 13.41; $p < 0.0001$; ANOVA: $F_{(3,154)}$= 27.02. Tukey's multiple comparison test: **$p < 0.01$, ***$p < 0.001$.

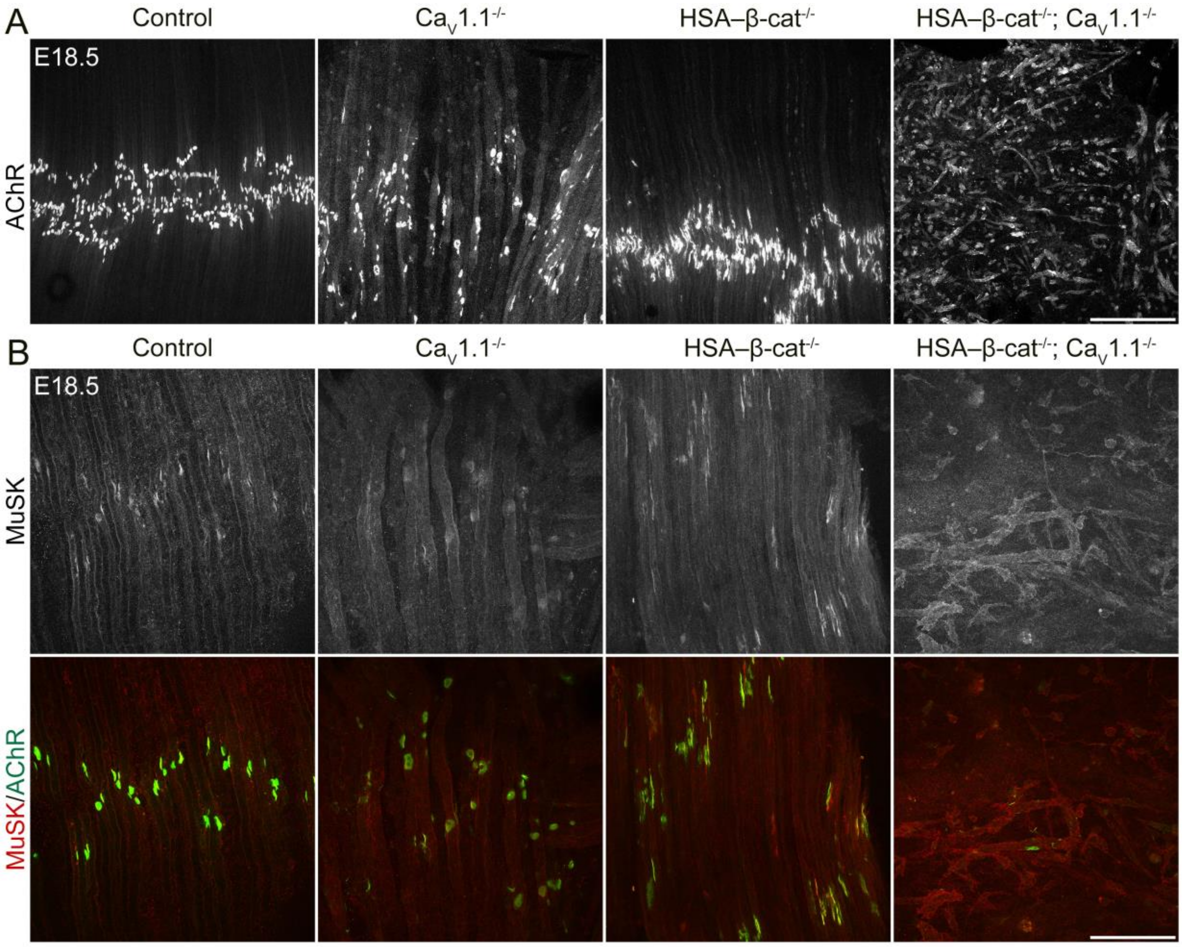

Supplemental Figure 3, related to Figure 3: Aberrant muscle fiber development in HSA–β-cat$^{-/-}$; Ca$_V$1.1$^{-/-}$ double-knockout mice. BTX **(A)** or BTX (green) and MuSK (red) **(B)** labeling of E18.5 diaphragms from control, Ca$_V$1.1$^{-/-}$, HSA–β-cat$^{-/-}$, and HSA–β-cat$^{-/-}$; Ca$_V$1.1$^{-/-}$ mice demonstrate abnormal muscle fiber development in double-knockout mice. BTX fluorescence brightness is strongly increased to indicate muscle fibers in **(A)**. Scale bars, 200µm for **(A)** and 100µm for **(B)**.
